# Mental Health Risks Among Informal Waste Workers in Kathmandu Valley, Nepal

**DOI:** 10.1177/00469580221128419

**Published:** 2022-10-18

**Authors:** Alisha Karki, Jiban Karki, Saugat Joshi, Michelle N. Black, Barsha Rijal, Srijana Basnet, Prabina Makai, Astrid Fossier Heckmann, Yuba Raj Baral, Andrew Lee

**Affiliations:** 1 PHASE Nepal, Kathmandu, Nepal; 2 University of Sheffield, Sheffield, UK; 3 Medecins du Monde, Saint-denis, Île-de-France, France; 4 Manmohan Memorial Institute of Health Sciences, Kathmandu, Nepal

**Keywords:** depression, informal waste worker, mental health, social protection, substance abuse

## Abstract

Informal waste workers are a vulnerable population group who are often socio-economically marginalized and disadvantaged, with more likelihood of experiencing ill health than the general population. To explore the determinants of mental ill health in this group, we conducted a cross-sectional survey of 1278 informal waste-workers in Nepal in 2017, using a demographic health assessment questionnaire and a modified Patient Health Questionnaire (PHQ-9). We looked at the potential associations between various exposure factors and mental health outcomes and found that 27.4% of waste-workers had depressive symptoms, more likely to be reported by female (OR 2.290), older person (OR 7.757), divorced/separated (5.859), and those with ill health (OR 2.030), or disability (OR 3.562). Waste-workers with access to social protection (OR 0.538) and financial savings (OR 0.280) were less likely to have depressive symptoms. There are key risk factors that may enable identification of particularly vulnerable persons within this group and also protective factors that may help improve their mental health resilience.

**What we already know**
• In Nepal, the living and working conditions of IWWs is poor and they have no access to social or physical infrastructure like basic sanitation, water, and power supply.
• Due to the lack of formal recognition and adequate representation, IWWs are largely isolated from most social-security schemes and legal protection frameworks.
**What this article adds**
• Waste-workers with access to social protection and financial savings were less likely to have depressive symptoms.
• There are protective factors that may help improve the mental health resilience of IWWs, such as social and financial protections, and greater social capital in the form of membership of co-operatives.

## Introduction

Waste is continuously generated by human activities. It is estimated that globally, approximately 2.01 billion tons of solid waste were produced in 2018 which is expected to grow to 3.40 billion tons by 2050.^1^ There are very few reliable estimates of the number of people engaged in collecting, sorting, and disposing of waste. Globally, it is estimated that around 56 million people (of whom 15 million are in developing countries) work in unhygienic and precarious conditions picking up, cleaning, sorting, and segregating recyclable waste.^2,3^ In India, there are approximately 4 million informal waste workers (IWW) who sell recyclable materials found on the streets to their local scrap dealers.^4^ In Nepal there is no definitive data on the number of IWWs, but a few studies have estimated that the numbers of IWWs in the Kathmandu valley is between 7000 and 15 000.^5^ As IWWs are a mobile population not limited to one geographical area, ascertaining an accurate estimation of their numbers is challenging.

Waste workers are likely to experience a significant amount of adverse health effects.^6^ Several studies undertaken in many developing countries including the Philippines, Brazil, and India have highlighted a wide range of occupational risks faced by informal waste workers such as chemical hazards, musculoskeletal damage, infection, risk of injury, emotional vulnerabilities, and environmental contamination.^7-10^ Common health issues reported include respiratory diseases, eye infections, stomach problems, typhoid fever, and diarrhea. Falls, accidents, waterborne diseases, and dermatological disorders are also prevalent among these groups.^6^ Female workers may also experience a high incidence of infections of the reproductive and urinary systems due to the lack of safe hygienic practices.^11^

The recycling activities carried out by IWWs makes a substantial contribution to society’s sanitary, environmental, and productivity conditions. Despite this, IWWs are often socially and economically marginalized around the world.^12^ Their work is frequently carried out in unhygienic and unsafe conditions, which are exacerbated by irresponsible waste disposal practices of both producers and consumers. Due to the lack of formal recognition and adequate representation, IWWs are largely isolated from most social-security schemes and legal protection frameworks.^13^ They often live apart from mainstream society and their precarious working conditions, low earnings and social stigma significantly contribute to an increased risk of psychological health issues.^14^ A study on the psychological well-being of IWWs in Mumbai for example found that shame, stigma, humiliation, and financial insecurity adversely affected their psychological health.^15^

In Nepal, the living and working conditions of IWWs is similarly poor. They have no access to social or physical infrastructure like basic sanitation, water, and power supply.^16^ IWWs are trapped at the bottom of the socioeconomic pyramid, and they face several challenges in their daily lives.^17^ They also have limited social security and social protection as state systems of social insurance usually do not cover informal workers in developing countries.^18,19^ Although some social protection schemes have been implemented in some areas in India,^20^ till now there is no similar scheme in Nepal apart from a few small initiatives that have sought to improve the well-being of IWWs through provision of health insurance, education, and development of entrepreneurial skills.^16^ We have previously reported the prevalence of symptoms of depression to be 27.4% in this group that is higher than the general population in Nepal.^21^ Consequently, measures to safeguard and improve the mental health and wellbeing of this disadvantaged group would be of benefit. However, there is limited robust information of the mental health and psychological wellbeing for this population group. This study therefore seeks to characterize and identify the possible determinants of mental health and wellbeing for IWWs in Nepal. In particular, we were interested to understand the possible links between socio-demographic status, substance abuse, social protections such as membership of a co-operative, and exposure to disaster (bearing in mind the recent Nepal earthquake in 2015), with depression.

## Methodology

### Study Design

A cross-sectional survey was conducted among informal waste workers (IWWs) in the Kathmandu valley and Nuwakot region between November and December 2017. This was part of a collaborative research project that sought to explore occupational risk mitigation and health status improvement for people working in informal waste collection, recycling, and trade in Nepal. The survey was targeted in the urban areas of Shantinagar and Teku in the Kathmandu valley, and Sisdole, Nuwakot, as these areas were known to have a high concentration of waste collection, processing sites, and dumpsites. As it was not possible to identify and recruit IWWs through random sampling, purposive, and snowball sampling was used. Known IWWs were invited to participate and snowball sampling was used to identify further IWWs to recruit to the study. Informed consent was received from the study participants whose participation was entirely voluntary. Full details of the methodology used for the survey have been previously published.^21^

### Sample Size

A convenience sampling was used. Study respondents were invited to participate by enumerators who visited the waste sites. Snowballing was then used to identify further respondents.^21^ A population estimate of 7000 IWWs was used for the total population size of this group in the Kathmandu valley based on previously reported figures.^5^ We assumed a 10% non-response rate and calculated that a sample size of 614 was needed to allow for a 4% level of precision, assuming a 50% prevalence of possible determinant variables, and a confidence level of 95%. We doubled the sample size to mitigate selection bias that may arise due to the non-probability sampling method used. This gave a total target sample size for this study of at least 1228 IWWs. In the end, 1278 IWWs were recruited in total.^21^

### Measurement Tools and Data Collection

A bespoke standardized demographic health assessment questionnaire^22^ was used for this survey for sociodemographic and health indicators. A modified Patient Health Questionnaire (PHQ-9) which was internationally validated,^23^ was used for screening depression among IWWs. The questionnaire was translated into the Nepali language. Pilot testing was done and necessary changes were made prior to data collection.

### Data Analysis and Management

Data cleaning and analysis were performed using IBM SPSS version 24. Descriptive analysis was presented using frequency and percentage. Crosstabulation was done between the independent variables and dependent variables. The main outcome indicator of interest was depression status as captured by the PHQ-9. This dependent variable (depression status) was dichotomized into “having depressive symptoms” and “having no depressive symptoms.” The Nepal PHQ9 was used for measuring depression status and has 5 categories based on scores (0-4 none, 5-9 mild, 10-14 moderate, 15-19 moderately severe, and 20-27 severe). For our dependent variable, a score of 0 to 4 was considered to have no depressive symptoms while scores between 5 and 27 were considered to indicate the presence of depressive symptoms among IWWs. Univariate and multivariate logistic regression was used to assess the association between the dependent and independent variables. The independent variables included socio-demographic variables, substance abuse, membership in co-operatives, having access to social protection or savings, presence of disability or ill health in the past 3 months, and whether they were affected by the Nepal earthquake disaster in 2015.

## Result

Table 1 presents the descriptive data of depression status among 1278 Informal Waste Workers (IWWs). As previously reported,^21^ 27.4% of the IWWs had depressive symptoms. Female IWWs (43.6%) were comparatively more likely to have depressive symptoms than male IWWs (23.5%). In terms of their country of origin, Nepali IWWs (30.4%) were more likely to report depressive symptoms compared to those from India (24.3%). Most of the IWW’s were between the ages of 25 and 39 years, and 28% of them reported having depressive symptoms. Older IWWs (above 55 years) were more likely to have depressive symptoms (57.1%) than other age groups.

**Table 1.** Depression Status Among Informal Waste Workers (IWWs) in Kathmandu Valley, Nepal (n = 1278).

Independent variables	Not having depressive symptoms (n = 927)	Having depressive symptoms (n = 350)	Total
Sex			
Male	770 (76.5%)	237 (23.5%)	1007
Female	145 (56.4%)	112 (43.6%)	257
Total	915 (72.4%)^*^	349 (27.6%)^*^	1264
Country of origin			
Indian	465 (75.7%)	149 (24.3%)	614
Nepali	461 (69.6%)	201 (30.4%)	662
Total	926 (72.6%)^*^	350 (27.4%)^*^	1276
Age			
18 to 24	296 (85.8%)	49 (14.2%)	345
25 to 39	440 (71.8%)	173 (28.2%)	613
40 to 54	165 (63.2%)	96 (36.8%)	261
55+	24 (42.9%)	32 (57.1%)	56
Total	925 (72.5%)^*^	350 (27.5%)	1275
Family living arrangement			
Living alone	382 (76.9%)	115 (23.1%)	497
Nuclear	320 (68.4%)	148 (31.6%)	468
Extended	58 (66.7%)	29 (33.3%)	87
Living with others (not family members)	79 (71.8%)	31 (28.2%)	110
Other	87 (77.0%)	26 (23.0%)	113
Total	926 (72.6%)^*^	349 (27.4%)^*^	1275
Marital status			
Single/Never married	209 (86.4%)	33 (13.6%)	242
Married	698 (70.5%)	292 (29.5%)	990
Divorced/separated	3 (37.5%)	5 (62.5%)	8
Widow/Widower	13 (39.4%)	20 (60.6%)	33
Total	923 (72.5%)^*^	350 (27.5%)	1273
Perception of risk at work			
Risky	660 (71.2%)	267 (28.8%)	927
Not risky	242 (76.1%)	76 (23.9%)	318
Total	902 (72.4%)^*^	343 (27.6%)^*^	1254
Literacy			
Literate	483 (76.3%)	150 (23.7%)	633
Illiterate	443 (68.9%)	200 (31.1%)	643
Total	926 (72.5%)^*^	350 (27.4%)	1276
Substance abuse			
Smoker			
Yes	356 (69.1%)	159 (30.9%)	515
No	570 (74.9%)	191 (25.1%)	761
Total	926 (72.6%)^*^	350 (27.4%)	1276
Number of cigarettes per day			
Not daily	4 (80.0%)	1 (20.0%)	5
<10	200 (67.3%)	97 (32.6%)	297
11 to 20	141 (73.4%)	51 (26.5%)	192
21 to 40	4 (30.7%)	9 (69.2%)	13
Total	349 (68.8%)^*^	158 (31.2%)^*^	507
Recreational drugs			
Yes	22 (62.9%)	13 (37.1%)	35
No	888 (72.7%)	334 (27.3%)	1222
Total	910 (72.4%)^*^	347 (27.6%)^*^	1257
Drink alcohol			
Yes	376 (70.8%)	155 (29.2%)	531
No	550 (73.3%)	195 (26.2%)	745
Total	926 (72.6%)^*^	350 (27.4%)	1276
Being a member of any co-operatives or group			
Not a member	834 (73.8%)	296 (26.2%)	1130
Member	92 (63.0%)	54 (37.0%)	146
Total	926 (72.6%)^*^	350 (27.4%)	1276

* Frequencies for separate categories may not add up to the overall sample size because of missing values.

40.3% of the participants were smokers, and 41.6% used alcohol. Recreational drug use was rare (2.8%). Nearly a third of IWWs who were smokers (30.9%) reported depressive symptoms. There was also a noticeable trend with those who smoked more being more likely to report depressive symptoms. Of the 35 IWWs who were drug users, 37% of them had depressive symptoms. Similarly, of the 531 IWWs who consumed alcohol, 29.2% had depressive symptoms. Membership of cooperatives was low. Out of 1278 IWWs, only 146 (11.4%) were members of any cooperatives or groups. Of these, 37.0% reported having depressive symptoms.

Logistic regression analysis was performed to find any association between the selected predictive variables with depression status (Table 2). The gender trends remained with female IWWs being twice as likely to have depressive symptoms compared to males. Similarly, age trends persisted with older IWWs (above 55 years of age) being nearly 8 times more likely to have depressive symptoms compared to young IWWs aged 18 to 24 years. Trends were also seen with depressive symptoms being more likely in those who were divorced/separated, and those who perceived their work to be risky. However, there were no statistically significant associations seen for family living arrangements, or literacy. The country of origin signal disappeared in the multivariate analysis.

**Table 2.** Association of Socio-Demographic Variables with Depression Status Among IWWs in Kathmandu Valley, Nepal (n = 1278).

Independent variables	Univariate analysis		Multivariate analysis	
	*P*-value	OR	*P*-value	Adjusted OR
Sex				
Male	1		1	
Female	.000	2.510 (1.885-3.3420	.000	2.290(1.579-3.321)
Age				
18 to 24	1		1	
25 to 39	.000	2.375 (1.674-3.370)	.003	1.933 (1.250-2.990)
39 to 54	.000	3.515 (2.372-5.208)	.000	3.012 (1.842-4.926)
55+	.000	8.054 (4.379-14.816)	.000	7.757 (3.665-16.417)
Family living arrangement				
Living alone	1		1	
Nuclear	.003	1.536 (1.155-2.044)	.719	0.939 (0.665-1.325)
Extended	.043	1.661 (1.015-2.717)	.856	1.054 (0.596-1.863)
Living with others	.264	1.303 (0.819-2.075)	.197	1.379 (0.847-2.246)
Other	.976	0.993 (0.611-1.613)	.798	1.071 (0.634-1.808)
Marital status				
Single	1		1	
Married	.000	2.649(1.791-3.920)	.330	1.287 (0.775-2.140)
Divorced/Separated	.002	10.556 (2.408-46.263)	.051	5.859 (0.992-34.598)
Widow/Widower	.000	9.744 (4.427-21.445)	.212	1.842 (0.706-4.806)
Country of origin				
Indian	1		1	
Nepali	.015	1.361 (1.062-1.744)	.438	1.113 (0.849-1.460)
Perception of risk at work				
Risky	1		1	
Not risky	.092	0.776 (0.578-1.042)	.033	0.703 (0.509-0.971)
Literacy				
Literate	1		1	
Illiterate	.006	1.654 (1.152-2.37)	.779	1.040 (0.789-1.371)

*Note.* OR = odds ratio.

In the univariate analysis for substance use (Table 3), our data indicated that smokers were 1.3 times more likely to have depressive symptoms compared to non-smokers. However, there was no significant associations seen with drug use or alcohol consumption in the multivariate analysis. Disability was significantly associated with having depressive symptoms. IWWs who reporting having any disability were 3 times more likely to have depressive symptoms compared to those with no disability. Similarly, IWWs who reported having ill health in the last 3 months were twice as likely to have depressive symptoms. There was no clear association with subjective reports of having been affected by the earthquake in 2015.

**Table 3.** Association of Different Variables With Depression Status Among IWWs in Kathmandu Valley, Nepal (n = 1278).

Association of substance abuse with depression status among IWWs				
Variables	Univariate		Multivariate	
	*P*-value	Unadjusted OR	*P*-value	Adjusted OR
Smoker				
Yes	.023	1.333 (1.039-1.709)	.062	1.290 (0.988-1.686)
No	1		1	
Drug use				
Yes	.204	1.571 (0.782-3.154)	.357	1.394 (0.688-2.825)
No	1		1	
Drink alcohol				
Yes	.234	1.163 (0.907-1.491)	.661	1.061 (0.813-1.385)
No	1		1	
Association of disability, affected by earthquake and ill health with depression status				
Disability				
Having disability	.000*	4.283 (3.300-5.559)	.000*	3.562 (2.719-4.668)
No disability	1		1	
Affected by earthquake				
Yes	.01	1.718 (1.235-2.388)	.173	1.280 (0.897-1.826)
No	1		1	
Ill health				
Yes	.000	2.779(2.151-3.589)	.000*	2.030 (1.546-2.666)
Can’t remember	.819	0.778(0.090-6.703)	.988	0.983 (0.108-8.943)
No	1		1	
Association of being a member of co-operative or groups, savings, and social protection with depression status				
Being member of co-operative or any group				
Not a member	.006	1.654 (1.152-2.373)	.016*	1.585 (1.088-2.311)
Member	1		1	
Social Protection				
Yes	.000	0.438 (0.275-0.697)	.012*	0.538 (0.330-0.874)
No	1		1	
Savings				
Yes	.000	0.276 (0.187-0.408)	.000*	0.280 (0.189-0.416)
No	1		1	

*Note.* CI = confidence interval; OR = odds ratio.

* *P*-value significant at .05 level.

IWWs who had access to some forms of social protection were less likely to have depressive symptoms compared to those who didn’t. Similarly, those IWWs who were members of any group or co-operatives were less likely to have depressive symptoms. Those who had financial savings were also less likely to have depressive symptoms compared to those who had no savings.

## Discussion

This study found that depressive symptoms were more likely to be reported by informal waste workers who were older, or were women. Possible protective factors identified included membership of any group or co-operative, access to social protection and savings. Risk factors included the presence of disability, or recent ill health, and the perception of their work being risky. There was no clear evidence of association between alcohol and drug use and depressive symptoms, although there was a weak association seen with smoking.

This finding is in line with several other studies on common mental health disorders in IWWs. A study undertaken in India with informal waste pickers observed that females were more vulnerable to mental health disorders compared to males.^24^ This may be because female waste workers experience more gender-based stressors in their work compared to their male counterparts. Likewise, a South African study on the prevalence of smoking and mental illness reported that informal waste pickers with mental illness are twice more likely to smoke.^15^

The financial vulnerability, low earnings and precarity of IWWs is well recognized and previous research has shown that waste workers risk being exploited socially and economically.^25^ Several studies have identified that the co-operatives and social protection schemes have immense potential to increase the social and economic inclusion of marginalized groups like IWWs in precarious working environments.^26^ In turn, this may help safeguard the psychological wellbeing of IWWs and protect against mental ill health. Our findings complements experience from India where many co-operatives have managed to secure a steady monthly salary for the waste pickers that in turn has secured the livelihoods of thousands of IWWs.^26^

In Nepal, several organizations are working to provide social recognition and protection to IWWs. In the context of Nepal, the recently adopted Labor Act, 2017 and Contributions Based Social Security Act, 2017 have promised to improve the rights of informal workers but so far that has not been effectively implemented.^27^ So far there are neither any plans or policies that include the social protection provision which could elevate the standing of the profession of the IWWs.

From several studies conducted worldwide, it is evident that IWWs have a poorer quality of life due to their stressful living and work conditions.^21^ IWWs will have a broad range of health and social needs, many of which are unlikely to be met. There will undoubtedly be a range of practical measures that can be implemented such health surveys, targeted health screening, as well as the provision of support, education and training opportunities. It is also apparent that the evidence base remains sparse and further studies, particularly focused on mental health and social protection, as well as potential effective interventions, are needed.

Amongst the limitations of this study are that the results may not be generalizable to all IWWs in Nepal as a whole, as the parent study was only undertaken in 2 districts. Moreover, participants in the survey were selected through snowball sampling methods, therefore it may not truly represent the entire community of informal waste pickers. Another limitation was there is a possibility that some useful information on the study aim could have been withheld by participants, although enumerators were well trained to minimize such effects. Since the survey had to be carried out in Nepali and Hindi languages, it could potentially lead to a subjective difference in the interpretation of the question by the participants. Finally, a recognized limitation of cross-sectional surveys is the inability to ascertain the temporal sequence and directionality of the associations observed.

## Conclusion

Poor mental health and wellbeing is not uncommon among informal waste workers and there are both key risk factors but also protective factors that may help mitigate. Decent working conditions, in both formal and informal sectors, could contribute to improve their mental health. Formalizing the waste work industry and increasing social protections may also substantially improve the psychological wellbeing of this vulnerable group.

## Recommendation

It is recommended to improve social protection for vulnerable groups like informal waste workers who rely on waste management for their livelihood. This study recommends the government to incorporate informal waste workers into social protection schemes, by improving their access to information and simplifying administrative procedures.

## Supplemental Material

sj-docx-1-inq-10.1177_00469580221128419 – Supplemental material for Mental Health Risks Among Informal Waste Workers in Kathmandu Valley, Nepal Click here for additional data file. Supplemental material, sj-docx-1-inq-10.1177_00469580221128419 for Mental Health Risks Among Informal Waste Workers in Kathmandu Valley, Nepal by Alisha Karki, Jiban Karki, Saugat Joshi, Michelle N. Black, Barsha Rijal, Srijana Basnet, Prabina Makai, Astrid Fossier Heckmann, Yuba Raj Baral and Andrew Lee in INQUIRY: The Journal of Health Care Organization, Provision, and Financing
