# Supplementary material for: Mental Health Risks Among Informal Waste Workers in Kathmandu Valley, Nepal
Source: Inquiry. 2022 Oct 18;59:00469580221128419. doi: 10.1177/00469580221128419 (PMC9580097; doi:10.1177/00469580221128419)
Supplement: sj-docx-1-inq-10.1177_00469580221128419 – Supplemental material for Mental Health Risks Among Informal Waste Workers in Kathmandu Valley, Nepal [file sj-docx-1-inq-10.1177_00469580221128419.docx]

**Survey of the Health Risks and Behaviours of Informal Waste Workers in the Kathmandu Valley, 2017**

**SURVEY QUESTIONNAIRE: PART A (for all respondents)**

| SN | Questions | Coding categories | Coding | Notes |
| --- | --- | --- | --- | --- |
| **SECTION A: SOCIO-DEMOGRAPHIC INFORMATION** | | | | |
| 1 | Country of birth | □ Nepal  □ India  □ Prefer not to say/Not known  □ Other (please specify:…………………………….) | 1  2  86  97 |  |
| 2 | Name of the district |  | *Free text* |  |
| 3 | Sex | □ Male  □ Female  □ Other (please specify:…………………………….) | 1  2  97 |  |
| 4 | Age (years) |  | *2 digits* |  |
| 5 | Religion | □ Hindu  □ Buddhist  □ Muslim  □ Kirat  □ Christian  □ Prefer not to say/Not known  □ Other (please specify:…………………………….) | 1  2  3  4  5  86  97 |  |
| 6 | Respondent's caste/ethnicity | □ Hill Dalit  □ Terai Dalit  □ Hill Janajati  □ Terai Janajati  □ Other Madeshi  □ Muslim  □ Brahmin/Chhetri  □ Prefer not to say/Not known  □ Other (please specify:…………………………….) | 1  2  3  4  5  6  7  86  97 |  |
| 7 | Family living arrangements | □ Living alone  □ Nuclear (father, mother, children)  □ Extended (above + grandparents)  □ Joint (including siblings of grandparents)  □ Living with others (not family members)  □ Other (please specify:…………………………….) | 1  2  3  4  5  97 |  |
| 8 | Types of accommodation | □ Own house  □ Rented house/room  □ Stay at the home of other family/friends  □ Stay at the temporary shelter  □ Sleep at the landfill site/ work site  □ No fixed abode/homeless  □ Other (please specify:…………………………….) | 1  2  3  4  5  6  97 |  |
| 9 | Have you ever attended school? | □ Yes  □ No | 1  2 | If No, go to Q11 |

| 10 | What is the highest grade finished? |  | *2 digits* |  |
| --- | --- | --- | --- | --- |
| 11 | What is your current marital status? | □ Single/Never married  □ Married  □ Divorced/separated  □ Widow  □ Widower  □ Other (please specify:…………………………….) | 1  2  3  3  4  97 |  |
| 12 | How many people live in your house? | ………….Male …………Female ………….Total | *2 digits* |  |
| 13 | Were you affected by the earthquake disaster?  (tick all that apply) | □ Not affected  □ Personally injured  □ Home was damaged/destroyed  □ Family members injured/killed  □ Lost livestock  □ Other (please specify:…………………………….) | 1  2  3  4  5  97 |  |

| **SECTION B: GENERAL HEALTH SURVEY** | | | | |
| --- | --- | --- | --- | --- |
| 1 | Have you been ill in last 3 months? | □ Yes  □ No  □ Can't remember | 1  2  3 | If No, go to next section |
| 2 | How many times have you been ill? | ……………. times in the last 3 months | *2 digits* |  |
| 3 | In the last four weeks did you have any of these symptoms? (multiple responses possible, tick all that apply) | □ Shortness of breath | 1 |  |
|  |  | □ Cough | 2 |  |
|  |  | □ Runny nose and frequently sneezing | 3 |  |
|  |  | □ Headache | 4 |  |
|  |  | □ Fever | 5 |  |
|  |  | □ Itching | 6 |  |
|  |  | □ Skin rashes | 7 |  |
|  |  | □ Painful, red or watery eyes | 8 |  |
|  |  | □ Dizziness | 9 |  |
|  |  | □ Nausea/vomiting | 10 |  |
|  |  | □ Abdominal pain | 11 |  |
|  |  | □ Diarrhoea (frequent loose stools) | 12 |  |
|  |  | □ Pain in arms and/or legs | 13 |  |
|  |  | □ Backache | 14 |  |
|  |  | □ Numbness in any part of body | 15 |  |
|  |  | □ Swelling of any body part | 16 |  |
|  |  | □ Injuries (e.g. cuts and bruises) | 17 |  |
|  |  | □ Burns | 18 |  |
|  |  | □ Tiredness | 19 |  |
|  |  | □ Heavy menstrual bleeding | 20 |  |
|  |  | □ Other (please specify:…………………………….) | 97 |  |

| **SECTION C : TOBACCO, DRUGS AND ALCOHOL** | | | | |
| --- | --- | --- | --- | --- |
| 1 | How many cigarettes do you smoke a day? | □ Don’t smoke  □ Less than 10 cigarettes a day  □ 11-20 a day  □ 21-40 a day  □ More than 40 a day | 1  2  3  4  5 |  |
| 2 | Do other people smoke in your house? | □ Yes  □ No | 1  2 |  |
| 3 | How many times a day do you chew tobacco (or take Khaini) | □ Don’t chew tobacco/use Khaini  □ Less than 5 times a day  □ 6-10 times a day  □ 11-20 times a day  □ More than 20 times a day | 1  2  3  4  5 |  |
| 4 | Do other people in your house chew tobacco or use Khaini? | □ Yes  □ No | 1  2 |  |
| **AUDIT C Screening Questions** | | | | |
| 5 | On average, how often do you drink alcohol? | □ Never (0 points)  □ Monthly or less (1 point)  □ 2-4 times a month (2 points)  □ 2-3 times a week (3 points)  □ 4 or more times a week (4 points) | 1  2  3  4  5 | If Never, go to Q7 |
| 6 | When you drink alcohol how much do you drink? | □ 1 or 2 drinks (0 points)  □ 3 or 4 drinks (1 point)  □ 5 or 6 drinks (2 points)  □ 7 or 8 or 9 drinks (3 points)  □ more than 10 drinks (4 points) | 1  2  3  4  5 |  |
| 7 | How often have you had 3 or more drinks if female, or 4 or more if male, on a single occasion in the last year? | □ Never (0 points)  □ Less than monthly (1 point)  □ Monthly (2 points)  □ Weekly (3 points)  □ Almost daily or every day 4 (points) | 1  2  3  4  5 |  |
| 8 | Do you use recreational drugs? | □ Yes  □ No | 1  2 |  |
| 9 | If yes ,what do you use? (tick all that apply) | □ Cannabis/Marijuana/Ganja  □ Heroin  □ Cocaine  □ LSD  □ Medical/Prescription drugs  □ Morphine  □ Glue sniffing  □ Other (please specify:…………………………….) | 1  2  3  4  5  6  7  97 |  |
| 10 | How often do you use the drugs? | □ Never  □ Monthly or less  □ 2-4 times a month  □ 2-3 times a week  □ 4 or more times a week | 1  2  3  4  5 |  |

| **SECTION D : HEALTH SERVICES** | | | | |
| --- | --- | --- | --- | --- |
| 1 | Are there any government health services in your area? | □ Yes  □ No  □ Don’t know | 1  2  3 |  |
| 2 | Where do you go for treatment when you are ill?  (tick all that apply) | □ Nearby government clinic  □ Government hospital  □ Private hospital  □ Private clinic  □ Private medicine shop / pharmacy  □ Traditional healer  □ Other (please specify:…………………………….) | 1  2  3  4  5  6  97 |  |
| 3 | How far is the health facility from here? | □ Less 30 minutes  □ 30-60 minutes  □ 1-2 hours  □ More than 2 hours  □ Don’t know | 1  2  3  4  5 |  |
| 4 | Are you satisfied with the services you received? | □ Yes  □ No  □ Do not want to comment | 1  2  3 | If Yes, go to Q6 |
| 5 | Why are you unhappy with service you received?  (tick all that apply) | □ Service was not good  □ Had to go elsewhere for tests  □ Had to go elsewhere for medicines  □ Service providers behaved badly  □ Waiting time was too long for check up  □ Cannot pay for the services  □ Other (please specify:…………………………….) | 1  2  3  4  5  6  97 |  |
| 6 | Do you treat yourself by buying medicines from the pharmacy? | □ Yes  □ No  □ Sometimes | 1  2  3 |  |
| 7 | If you have children, have they been vaccinated? | □ Yes  □ No  □ Don’t know  □ No children | 1  2  3  4 | If No children, go to next section |
| 8 | If yes, where did you take them for their vaccinations? | □ Government health post  □ Private clinic  □ Government hospital  □ Other (please specify:…………………………….) | 1  2  3  97 |  |

| **SECTION E: MENTAL HEALTH AND WELLBEING** | | | | | | | |
| --- | --- | --- | --- | --- | --- | --- | --- |
| 1 | Have you had heart-mind problems in the past 2 weeks, for example thoughts playing in your heart-mind, sadness in your heart-mind, or worry in your heart-mind? | □ Yes  □ No | | | | 1  2 | If No, go to next section |
| 2 | During the past 2 weeks, have you experienced problems in your work, taking care of yourself and your family, or in your relationships with other people because of the problems that we talked about heart-mind problems? | □ Yes  □ No | | | | 1  2 | If No, go to next section |
|  | **Nepal PHQ 9 Depression Questions** | **Not at all (0)** | **Some-times (1)** | **Usually**  **(2)** | **Always**  **(3)** | |  |
| 3 | During the past 2 weeks, compared to other people, how much have you felt that you are not able to be happy or do not enjoy doing work/activities? | □ | □ | □ | □ | |  |
| 4 | During the past 2 weeks, how much have you felt frustrated, despairing or incapable of doing anything? | □ | □ | □ | □ | |  |
| 5 | During the past 2 weeks, how much have you had problems with your sleep, such as not being able to sleep properly and peacefully, or feeling sleepier than before? | □ | □ | □ | □ | |  |
| 6 | During the past 2 weeks, how much have you felt tired and lacking energy? | □ | □ | □ | □ | |  |
| 7 | During the past 2 weeks, how much have you lost your appetite or experienced increased appetite? | □ | □ | □ | □ | |  |
| 8 | During the past 2 weeks, how much have you blamed yourself for something or felt that you have let you or your family down? (For example, because of you, you and your family have lost respect in the society?) | □ | □ | □ | □ | |  |
| 9 | During the past 2 weeks, how much have you been having difficulty being able to focus or concentrate? (For example, not being able to concentrate while watching TV, reading a newspaper, cleaning rice, cooking, or working?) | □ | □ | □ | □ | |  |
| 10 | During the past 2 weeks, how much have people commented that you have been talking very softly, walking slowly, moving around needlessly or acting restless? | □ | □ | □ | □ | |  |
| 11 | During the past 2 weeks, how much have you had the feeling of hurting yourself, dying or committing suicide? (For example, cutting your hands, taking poison, jumping from some-where, hitting your head against the wall?) | □ | □ | □ | □ | |  |
|  | TOTAL SCORE (Q3+4+5+6+7+8+9+10+11) |  | | | | |  |

| **SECTION F: SEXUAL & REPRODUCTIVE HEALTH** | | | | |
| --- | --- | --- | --- | --- |
| 1 | Do you know about family planning methods? | □ Yes  □ No | 1  2 |  |
| 2 | Did you know that condoms can help prevent HIV and infections? | □ Yes  □ No | 1  2 |  |
| 3 | If you are in a sexual relationship, are you or your partner using any methods to prevent pregnancy or STIs? | □ Yes  □ No  □ Don’t know | 1  2  3 | If No, go to Q5 |
| 4 | Which method are you (or your partner) using? (Don’t prompt, tick all that apply) | □ Female sterilization  □ Male sterilization  □ Intrauterine device (IUD)  □ Injectable  □ Implant  □ Condom  □ Pills  □ Rhythm method  □ Withdrawal  □ Other (please specify:…………………………….) | 1  2  3  4  5  6  7  8  9  97 |  |
| 5 | In the last 3 years have you (or has your wife) been pregnant or given birth to a baby? | □ Yes  □ No | 1  2 | If No, go to Q9 |
| 6 | Did you/she have antenatal clinic (ANC) checkup when you were / she was pregnant last time? | □ Yes  □ No  □ Can’t remember/don’t know | 1  2  3 | If No, go to Q8 |
| 7 | How many times did you / she have ANC checkup? | …………….times |  |  |
| 8 | Did you / she have postnatal (PNC) check up after birth? | □ Yes  □ No  □ Can’t remember/don’t know | 1  2  3 |  |
| 9 | Did you / she have any miscarriage or abortion during last three years? | □ Yes  □ No  □ Can’t remember/Don’t know | 1  2  3 |  |

| **SECTION G: DISABILITY SCREENING** | | | | |
| --- | --- | --- | --- | --- |
| 1 | Do you have difficulties with seeing (even if wearing glasses)? | □ No - no difficulty  □ Yes – some difficulty  □ Yes – a lot of difficulty  □ Cannot do at all | 1  2  3  4 |  |
| 2 | Do you have difficulties with hearing (even with a hearing aid) | □ No - no difficulty  □ Yes – some difficulty  □ Yes – a lot of difficulty  □ Cannot do at all | 1  2  3  4 |  |
| 3 | Do you have difficulties with Walking or climbing steps | □ No - no difficulty  □ Yes – some difficulty  □ Yes – a lot of difficulty  □ Cannot do at all | 1  2  3  4 |  |
| 4 | Do you have difficulties with remembering or concentrating | □ No - no difficulty  □ Yes – some difficulty  □ Yes – a lot of difficulty  □ Cannot do at all | 1  2  3  4 |  |
| 5 | Do you have difficulties with self care, such as washing all over or dressing | □ No - no difficulty  □ Yes – some difficulty  □ Yes – a lot of difficulty  □ Cannot do at all | 1  2  3  4 |  |
| 6 | Do you have difficulties with communicating (using your usual language), e.g. understanding or being understood | □ No - no difficulty  □ Yes – some difficulty  □ Yes – a lot of difficulty  □ Cannot do at all | 1  2  3  4 |  |
| 7 | Do you look after someone with (like what we’ve just talked about)? | □ Yes  □ No | 1  2 |  |
| 8 | Do you look after someone with chronic health problems? | □ Yes  □ No | 1  2 |  |

| **SECTION H: SOCIAL SECURITY** | | | | |
| --- | --- | --- | --- | --- |
| 1 | Are you a member of any groups or cooperatives? | □ Yes  □ No | 1  2 | If No, go to Q3 |
| 2 | What groups or cooperative are you involved with? | □ Cooperative  □ Mothers group  □ Community groups  □ Local clubs  □ Saving or credit groups  □ Other (please specify:…………………………….) | 1  2  3  4  5  97 |  |
| 3 | Do you receive any of these social protections? | □ Health insurance  □ Free education for kids  □ Accident insurance  □ Any emergency fund  □ Other (please specify:…………………………….) | 1  2  3  4  97 |  |

| **SECTION I : EMPLOYMENT & FINANCIAL** | | | | |
| --- | --- | --- | --- | --- |
| 1 | What is your occupation  (multiple responses possible, tick all that apply) | □ Agriculture  □ Waste collection  □ Waste sorting  □ Waste dealer  □ Student  □ Business  □ Not working/unemployed  □ Housewife/caring for family  □ Other (please specify:…………………………….) | 1  2  3  4  5  6  7  8  97 |  |
| 2 | Does anyone in your family work as a waste worker? | □ Yes  □ No  □ Don’t know/prefer not to say | 1  2  3 |  |
| 3 | How many people in your family work as a waste worker? | ………….Male …………Female …………Total | *2 digits* |  |
| 4 | Does your spouse also work as a waste worker? | □ Yes – often/all the time  □ Yes – sometimes/rarely  □ No  □ Not relevant as not married/no spouse  □ Don’t know/prefer not to say | 1  2  3  4  5 |  |
| 5 | Do any of your children work as waste workers? | □ Yes – often/all the time  □ Yes – sometimes/rarely  □ No  □ Don’t know/prefer not to say | 1  2  3  4 |  |
| 6 | Do you have any debts or loans? | □ Yes  □ No  □ Don’t know/prefer not to say | 1  2  3 | If No, go to Q7 |
| 7 | If you have debts or loans, who are the lenders?  (tick all that apply) | □ Neighbour/friend  □ Scrap dealers  □ Cooperative  □ Bank  □ Other (please specify:…………………………….) | 1  2  3  4  97 |  |
| 8 | If you have savings, where do you deposit your savings? | □ Keep at home  □ Scrap dealers  □ Bank  □ Cooperative  □ Other (please specify:…………………………….) | 1  2  3  4  97 |  |
| 9 | Are there times of the year that you do not have work? | □ Yes  □ No | 1  2 |  |
| 10 | If yes, how many months a year | …………………… months | *2 digits* |  |

| **SURVEY QUESTIONNAIRE: PART II (for respondents who work as informal waste workers)**  *The following section is only to be completed for respondents who are informal waste workers* | | | | |
| --- | --- | --- | --- | --- |
| **SECTION J: IWW PROFILE** | | | | |
| 1 | How long have you worked as a WW? | ………….. years | *2 digits* |  |
| 2 | What waste do you collect? | □ Plastic bottles  □ Plastic bags  □ Papers  □ Aluminium  □ Electronic goods  □ Glass  □ Cloth/fabric  □ Copper  □ Other (please specify:…………………………….) | 1  2  3  4  5  6  7  8  97 |  |
| 3 | Why do you work as WW?  (tick all that apply) | □ This is an easy job / easy to earn money  □ No need for any initial investment  □ Don’t own land for work  □ This is a family business  □ Work place is near to my house  □ Able to work independently  □ Suggested by family and friends  □ No other work available  □ Other (please specify:…………………………….) | 1  2  3  4  5  6  7  8  97 |  |
| 4 | How many hours do you work per day? | ……………..hrs/per day | *2 digits* |  |
| 5 | How many days do you work per month? | ………………days/ month | *2 digits* |  |
| 6 | Do you like this job? | □ Yes  □ No | 1  2 |  |
| 7 | How long do you think you will do this job for? | □ Just for a few weeks or months (short term)  □ For a few years (medium term)  □ For many years (long term)  □ Don’t know | 1  2  3  4 |  |
| 8 | How much waste do you collect in a day? | ……………….. kg/day | *3 digits* |  |
| 9 | What do you do with the waste you collect?  (tick all that apply) | □ Sell them on  □ Collection only  □ Sorting  □ Dismantling  □ Metals recovery  □ Burning  □ Re-use them  □ Other (please specify:…………………………….) | 1  2  3  4  5  6  7  97 |  |
| 10 | How much money do you earn per day from WW? | …………………….. NPR/day | *4 digits* |  |
| 11 | How much money do you save per day from WW? | …………………….. NPR/day | *4 digits* |  |

| **SECTION K: KNOWLEDGE OF HEALTH RISKS OF WASTE WORK** | | | | |
| --- | --- | --- | --- | --- |
| 1 | Do you think waste work is a risky job | □ Yes  □ No  □ Don’t know | 1  2  3 |  |
| 2 | How much risk do you think you have doing this job? | □ High risk  □ Low risk  □ Don’t know | 1  2  3 |  |
| 3 | Have you ever received any information about the risks of waste work? | □ Yes  □ No  □ Can’t remember | 1  2  3 | If No, go to Q5 |
| 4 | Where did you get that information from? | □ School  □ Neighbour/family/friends  □ Government organization  □ INGO  □ NGO  □ Cooperative  □ Can’t remember  □ Other (please specify:…………………………….) | 1  2  3  4  5  6  7  97 |  |
| 5 | What do you think are the possible health risks of working as a WW? (do not prompt, tick all that apply) | □ Don’t know  □ Bad for lungs/causes breathing problems  □ Risk of injury  □ Risk of infection  □ Bad for eyes  □ Skin diseases  □ Headache  □ Mental stress / health problem  □ Other (please specify:…………………………….) | 1  2  3  4  5  6  7  8  97 |  |
| 6 | How do you think you can protect yourself from the risks of waste work?  (tick all that apply) | □ Don’t know  □ Use gloves  □ Wearing masks  □ Wearing safety boots  □ Washing hands before eating  □ By not eating at the work place  □ Drinking clean water  □ Changing your clothes after work  □ Other (please specify:…………………………….) | 1  2  3  4  5  6  7  8  97 |  |

| **SECTION L : PERSONAL PROTECTION** | | | | |
| --- | --- | --- | --- | --- |
| 1 | Do you change your clothes immediately after work? | □ Yes, always  □ Sometimes  □ No | 1  2  3 |  |
| 2 | Do you shower or clean your body after work? | □ After work immediately at the work place  □ After returning back home  □ Do not shower or clean every day | 1  2  3 |  |
| 3 | Do you wash your hands before eating your lunch when you are at work? | □ Yes, always  □ Sometimes  □ No | 1  2  3 | If Yes, go to Q5 |

| 4 | If you do not wash your hands before meals, why is that? | □ No water available  □ No need to wash  □ Eat with spoon  □ Not my habit to wash hand  □ Other (please specify:…………………………….) | | | | | 1  2  3  4  97 |  |
| --- | --- | --- | --- | --- | --- | --- | --- | --- |
| 5 | What personal protective equipment (PPE) do you use for work?  (tick all that apply) | □ None | | | | | 1 |  |
|  |  | *Frequency of PPE use* | *Always* | *Some times* | *Rarely* | *Never* |  |  |
|  |  | a) Glove | □ | □ | □ | □ | 2 |  |
|  |  | b)Apron | □ | □ | □ | □ | 3 |  |
|  |  | c) Cap/Net | □ | □ | □ | □ | 4 |  |
|  |  | d) Facemask | □ | □ | □ | □ | 5 |  |
|  |  | e) Glasses/Goggles | □ | □ | □ | □ | 6 |  |
|  |  | f) Boot/shoes | □ | □ | □ | □ | 7 |  |
|  |  | g) Helmets | □ | □ | □ | □ | 8 |  |
|  |  | h) Hi-visibility jacket | □ | □ | □ | □ | 9 |  |
|  |  | i) Other protection | □ | □ | □ | □ | 97 |  |
|  | If other protection used, please specify |  | | | | |  |  |

| **SECTION M : HEALTH CONSEQUENCES OF WASTE WORK** | | | | |
| --- | --- | --- | --- | --- |
| 1 | In the last 12 months, have you experienced any injuries at work?  (Multiple responses possible, tick all that apply) | □ No injury  □ Can’t remember  □ Metal cut  □ Glass cut  □ Needlestick injury  □ Hit by the truck/van or other vehicle  □ Fall during waste collection  □ Animal bite  □ Other (please specify:…………………………….) | 1  2  3  4  5  6  7  8  97 |  |
| 2 | How many times were you injured? | ……………. Times | *2 digits* |  |
| 3 | Have you experienced any kind of violence at work?  (Multiple responses possible, tick all that apply) | □ Not experienced any violence  □ Sexual harassment  □ Physical violence  □ Verbal abuse  □ Rape  □ Other (please specify:…………………………….) | 0  1  2  3  4  97 |  |

Thank you very much for taking part and providing valuable information.
